# Determination of the relative configuration of tropinone and granatanone aldols by using TBDMS ethers

**DOI:** 10.3762/bjoc.8.216

**Published:** 2012-11-02

**Authors:** Ryszard Lazny, Aneta Nodzewska, Katarzyna Sidorowicz, Przemyslaw Kalicki

**Affiliations:** 1 Institute of Chemistry, University of Bialystok, ul. Hurtowa 1, 15-339 Bialystok, Poland; 2 Institute of Organic Chemistry, Polish Academy of Sciences, ul. Kasprzaka 44/52, 01-224 Warsaw, Poland

**Keywords:** aldol reaction, *exo*–*endo* isomerization, granatanone, stereoselective reaction, tropinone

## Abstract

The relative configurations of *tert*-butyldimethylsilyl (TBDMS) ethers of all four diastereomers of the aldols of tropinone (8-methyl-8-azabicyclo[3.2.1]octan-3-one), as well as of granatanone (9-methyl-9-azabicyclo[3.3.1]nonan-3-one), were determined from NMR data, and from the observed interconversion of the diastereomers (*exo*,*anti* to *endo*,*syn* and *exo*,*syn* to *endo*,*anti*). The *exo* forms invert to *endo* isomers in the presence of silica gel. The relative configuration of a new isomer of tropinone aldol accessible synthetically through the direct solventless reaction of tropinone and benzaldehyde in the presence of water was determined as *exo*,*syn* by comparison of NMR data of the aldol isomers, in particular vicinal coupling constants and shifts corresponding to the side-chain CH group, with data of related TBDMS derivatives and confirmed by single-crystal X-ray diffraction.

## Introduction

**Figure 1:**
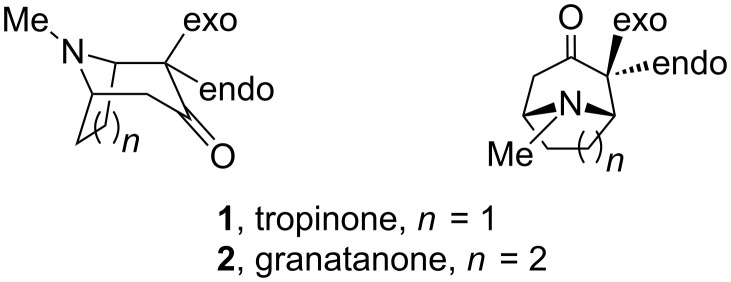
The *exo* and the *endo* substituents at the α-carbon (C-2 by tropane numbering) of the tropinone or granatanone scaffold shown in two alternative projections.

**Scheme 1:**
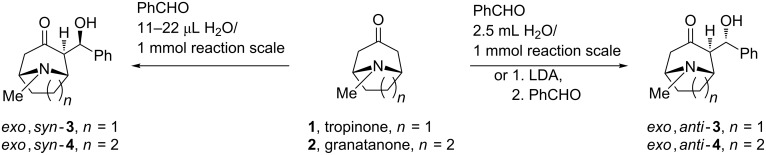
Typical preparation of representative tropinone and granatanone aldols [3,17,20].

Enantiomerically pure, and racemic, diastereomerically pure aldols of tropinone have been used as key intermediates in stereoselective syntheses of natural tropane alkaloids and their analogues [1], including the unnatural enantiomer of cocaine (*ent*-cocaine) [2], knightinol [3], alkaloid KD-B [3] and ferrugine [4–5]. Stereoselective syntheses of nortropinone aldols [6–7] and N-protected nortropinone aldols [5,8–9], which can open access to other N-substituted analogues, have also been described. The known diastereomerically and enantiomerically pure aldols of tropinone (8-methyl-8-azabicyclo[3.2.1]octan-3-one) and granatanone (9-methyl-9-azabicyclo[3.3.1]nonan-3-one) (Figure 1) were synthesized by enantioselective deprotonation with lithium amide bases followed by diastereoselective aldol reaction with aldehydes [10–11]. In all the aldol reactions promoted by achiral (LDA [5,12]) or by chiral lithium amides (e.g., lithium bis(1-phenylethyl)amide [4–5] or Koga’s amide [3]), including amides immobilized on a polymeric carrier [13–14], the major products had the *exo*,*anti* configuration (Scheme 1).

Configuration of the *exo*,*anti*-**3** was assigned based on X-ray diffraction [15]. Granatanone aldols, including *N*-benzyl derivatives, obtained with lithium amides were identified as the *exo*,*anti* isomers based on X-ray crystallography [16–17]. Granatanone aldols with *exo*,*syn* configuration have been observed only as minor products [17]. Until recently other isomers and synthetic procedures for their preparation remained unknown. We succeeded in preparing other isomers through an unexpected direct reaction of the ketones with aromatic aldehydes in the presence of catalytic amounts of water (Scheme 1) [18–20]. In our preliminary synthetic report the major isomeric products accessible under these conditions were assigned the *exo*,*syn* configuration. DFT calculations suggest that selectivity in the solventless reaction results from equilibration and the higher stability of the O–H^…^N-hydrogen-bond-stabilized conformer of the *exo*,*syn* isomer [20–21].

Herein, we wish to report a method used for identification of the relative configuration of this new isomer of the tropinone aldol and closely related granatanone aldol. To the best of our knowledge, all diastereoisomers of tropinone and granatanone aldols synthesized to date have the *exo* configuration. The other possible isomers, i.e., the *endo* forms, have never been isolated or described. Their appearance in some experiments has been mentioned [22]. We have made an effort to devise a way to identify these elusive *endo* isomers.

In this paper, we propose a simple method for the fast identification of all of the diastereomers of tropinone and granatanone aldols, including the *endo* isomers, through their silyl ether derivatives.

## Results and Discussion

The new aldol product of the solventless reaction of tropinone with benzaldehyde had relative stereochemistry different from the *exo*,*anti* configuration, as judged from its NMR spectra. In particular the chemical shift and coupling constant of the characteristic carbinol C*H* signal differed from the known *exo*,*anti* isomer. Coupling constants of both isomers (3.1 Hz for *exo,anti* and 2.6 Hz for the new product) were similar. Usually the vicinal coupling constants for *syn* and *anti* isomers differ enough to allow for configuration assignment [23] (e.g., aldols of cyclic ketones such as piperidone and cyclohexanone fall within the following typical ranges: *syn*: ca. 2–3 Hz, *anti*: ca. 7–9 Hz).

**Scheme 2:**
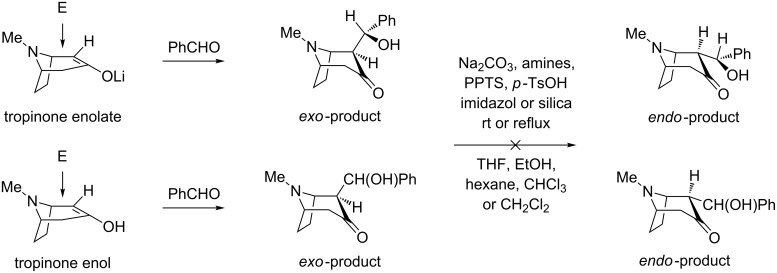
Sterically and stereoelectronically favored trajectory for the approaching electrophile (E) and attempted inversions of configuration of the known and unknown tropinone aldol isomers.

In principle the new product could have had any of the three remaining possible configurations. However, we suspected that the unknown isomer was likely the *exo*,*syn*-aldol (i.e., the other *exo* product formed by stereoelectronically and sterically favored axial attack) [24] or the *endo*,*syn* isomer (resulting from the *exo*–*endo* inversion of the known *exo*,*anti*-**3**, Scheme 2).

The question of whether the *exo* isomer could invert to the *endo* form was answered by attempted isomerization (Scheme 2) under various conditions (basic, acidic, imidazole, silica gel in various solvents). The *exo*,*anti* aldols showed only decomposition to tropinone and benzaldehyde or elimination to benzylidene derivatives. The behavior of the new isomer under the same isomerization conditions was similar and gave no indications regarding its stereochemical form. It appeared that in this case (as it was for the *exo*,*anti* isomer [3]) attempts to convert the presumed *exo* isomers to the expectedly more thermodynamically stable *endo* isomers would be also fruitless, unless the hydroxy group was protected (e.g., by silyl ether). In light of the sensitive nature of the aldols we reasoned that making a stable derivative was necessary.

**Scheme 3:**
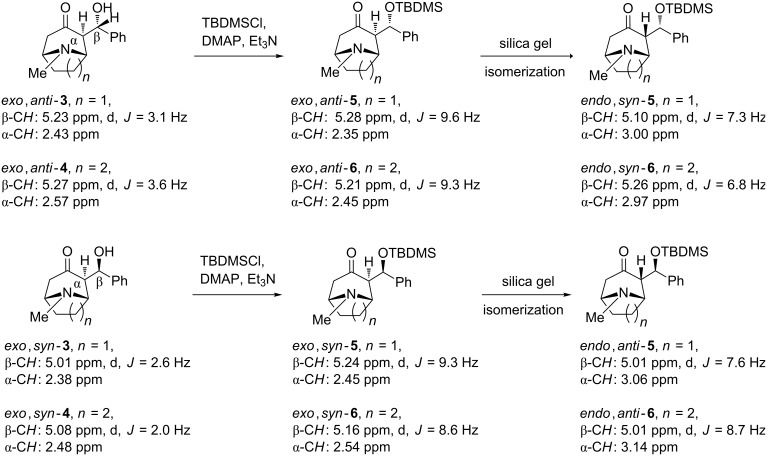
Preparation of TBDMS derivatives of all diastereomers of tropinone and granatanone aldols (included are characteristic NMR data for the side-chain β-C*H* protons and shifts of the axial or equatorial α-C*H* protons (at C-2)).

The TBDMS ethers of aldols have been already used for *exo*–*endo* isomerization of tropinone aldols [3]. Blocking of the hydroxy group with an ether or ester group would make formation of internal hydrogen bonding to the amine nitrogen or the carbonyl oxygen H-bond acceptors impossible, thus changing significantly the structure of aldols in aprotic solvents used for NMR. *N*-Benzyl analogues of the granatanone [16] and tropinone [9] aldols as well as aldols of granatanone [17] showed in crystalline form an intramolecular hydrogen bonding involving the free hydroxy group. We expected that such a change, reflected in the chemical shift and couplings, could be useful for discerning relative configurations. The aldol of unknown configuration was converted into silyl ether by reaction with *tert*-butyldimethylsilyl chloride (TBDMSCl) in the presence of DMAP and triethylamine (Scheme 3).

Because TBDMS ethers **5** with *exo*,*anti* and *endo*,*syn* configuration, as well as a method for their conversion, has been described [3], we expected that through comparison of ^1^H NMR data we would be able to exclude or confirm the structure of the new aldol TBDMS ether as *endo*,*syn.* The TBDMS derivative was different from the known *endo*,*syn*-isomer **5**. Thus the new aldol was either the *exo*,*syn* or the *endo*,*anti* isomer. Inducing isomerization of the unknown aldol TBDMS ether to the typically more stable *endo* form could be an indication pointing to the *exo*,*syn* configuration. From the tested methods for isomerization, the most suitable turned out to be the absorption on a silica column combined with separation. Although the silyl ether of the new isomer of tropinone aldol isomerised in the presence of silica, giving the last of the four isomeric TBDMS derivatives, in a similar way as described for the *exo*,*anti*-**5**, its conversion was noticeable faster than for the *exo*,*anti*-**5**. At this point it was clear that the new aldol isomer must have had the *exo*,*syn* configuration. Isomerization of its TBDMS derivative was so facile that its chromatographic purification was hardly possible. This agreed with expectations based on intuitive van der Waals radii and conformational analysis. Inspection of molecular models indicates that the pseudoaxial substituent of the *syn* isomer should experience more steric congestion on the tropinone bicyclic skeleton with equatorial *N*-methyl [9], than the same group of the *anti* configuration. It was interesting to see if one could obtain the free aldols in the *endo* forms by TBDMS deprotection. Under the typical desilylation conditions (1 M TBAF in THF) no formation of aldols, but rather products of their decomposition were detected in the resulting complex reaction mixtures. The presence of a relatively acidic pseudoaxial hydrogen atom at the α-carbon to carbonyl (C-2) is likely responsible for the facile elimination of a water molecule and formation of an isomeric mixture of condensation products ((*E*) and (*Z*)-2-benzylidene tropinones) in analogy to the known elimination of acetic acid from the acetyl derivative of the aldol [3].

More conclusive indication came from comparison of the characteristic signals (doublets) of the side-chain C*H* groups (side-chain β-carbon to the carbonyl group, Scheme 3). The fact that ^1^H NMR signals of *exo*,*syn*-**5** differed markedly from the *endo*,*syn*-**5** (as compared on the same NMR instrument) but were similar to the *exo*,*anti*-**5** supported the tentative assignment further. Knowing that in tropinone the axial α-protons are deshielded versus the equatorial protons by ca. 0.5 ppm [12,25], we were also able to identify positively, within the pairs of interconverting isomeric TBDMS ethers, the isomer with *exo* and *endo* side chain (equatorial and axial α-CH, respectively). On the basis of the observed *exo*–*endo* isomerization, and trends in NMR data changes upon isomerization, we were fairly certain of the assigned stereochemistry of the new isomer as *exo*,*syn*-**3**. This procedure developed on tropinone aldols was reproduced on corresponding granatanone aldols, providing TBDMS ethers of all four possible diastereomers of **6** (Scheme 3) and identifying the major product of the solventless reaction of granatanone with benzaldehyde [20–21] as *exo*,*syn*-**4**.

**Figure 2:**
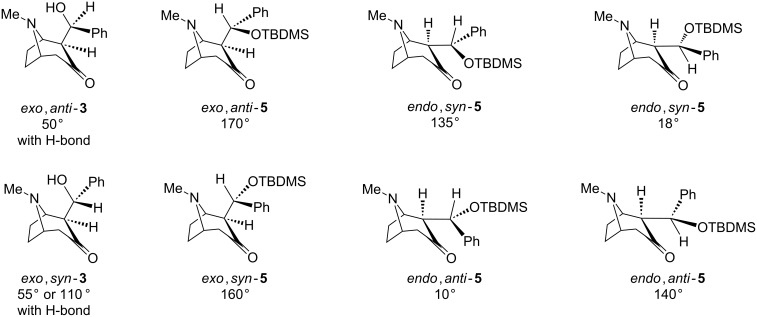
Approximate representations of likely conformations of tropinone aldols and their TBDMS ethers in solution, having dihedral H–αC–βC–H angles accounting for the observed vicinal couplings.

The vicinal coupling constants characteristic for the aldols and the TBDMS ethers (Scheme 3) can provide some insight into the preferred conformations in solution. Using the familiar Karplus correlation of the vicinal *J* and the dihedral angle [26–28], we suggest the preferred conformations shown in Figure 2. The aldol conformations shown could likely be stabilized by formation of an internal H-bond to the amine nitrogen as observed in crystals.

The dihedral angle in the structure *exo*,*anti*-**3** is estimated, based on *J* = 3.1 Hz, to be ca. 50° and for *exo*,*syn*-**3** slightly more than 55° or 110°, based on *J* = 2.6 Hz. The alternative conformations, without a possible H-bond to nitrogen for *exo*,*anti*-**3**, and an angle of ca. 110–115° corresponding to *J* = 3.1 Hz would have significant steric strain. In both TBDMS derivatives *exo*,*anti*-**5** and *exo*,*syn*-**5** the estimated dihedral angle of 160–170° corresponds to reasonable conformations. In the *endo* forms **5** the angle determined as 18° or 135° for *endo*,*syn*-**5** and 10° or 140° for *endo*,*anti*-**5** can be found in two of the shown alternative arrangements. The analysis for tropinone aldols is representative also for the granatanone analogues **4** and **6**.

**Figure 3:**
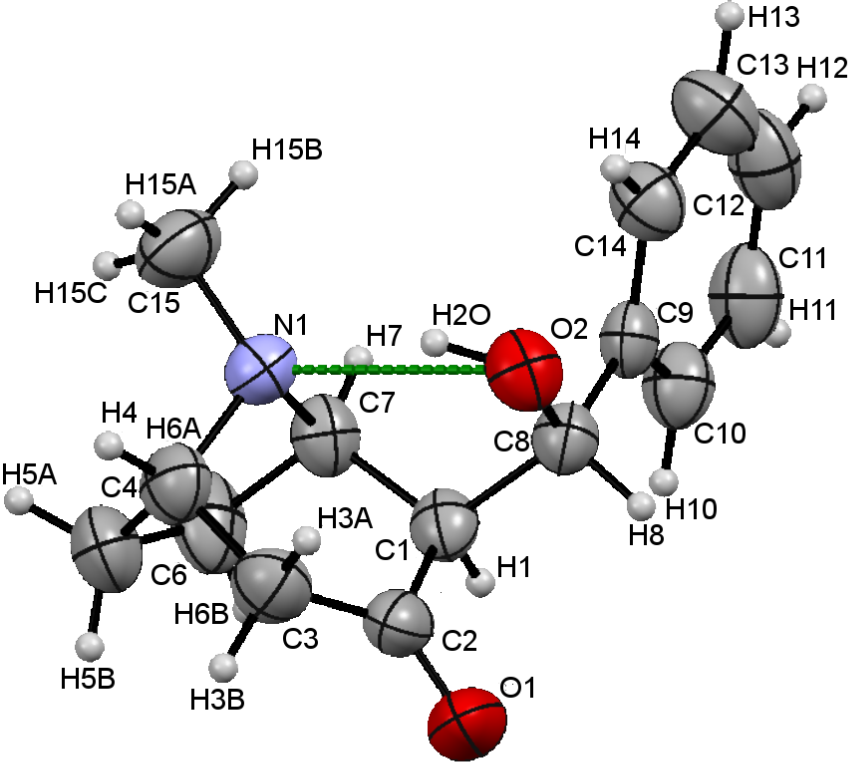
X-ray structure of aldol *exo*,*syn*-**3** synthesized by direct, solventless reaction of tropinone with benzaldehyde in the presence of catalytic amounts of water, showing an intramolecular H-bond: N1^…^O2 2.763(2), N1^…^H 1.920(2) Å, N1^…^H2O^…^O2 angle 151(2)°. Displacement ellipsoids are drawn at 30% probability level; atom numbering is arbitrary.

After identifying the *exo*,*syn* configuration by the described procedure we succeeded in preparing the crystalline form of the new isomer of **3** suitable for single crystal diffraction. Meticulous purification by precipitation with hexane, followed by slow crystallization from ether gave suitable crystals of the isomer, configuration of which was unambiguously determined as *exo*,*syn* (Figure 3) by X-ray diffraction.

Atoms H1 and H8 were found in *syn* relation to each other, while the 1′-hydroxybenzyl group at C1 is on the *exo* side of the bicyclic tropane scaffold pointing towards the C4–N1–C7 bridge (pseudoaxial on the tropinone six-membered ring). The crystal structure is also characterized by internal hydrogen bonding between the nitrogen N1 and the oxygen O2 atom (N1–H2O–O2). The configuration of a related granatanone aldol (*p*-NO_2_-PhCHO derived [20]) was also confirmed by X-ray analysis as *exo*,*syn* with analogous hydrogen bonding [17].

## Conclusion

All four diastereomers of the aldols of tropinone and granatanone with benzaldehyde were characterized as TBDMS ethers. Assignment of the configuration was possible on the basis of comparison of the chemical shifts and coupling constants relevant to the side chain of the TBDMS ethers. The *endo* isomers could be prepared by equilibration combined with separation on a silica column. The major products of the direct solventless aldol reaction of tropinone and granatanone [20] were assigned the *exo*,*syn* configuration on the bases of their NMR data and NMR data of the corresponding TBDMS ethers. The assigned *exo*,*syn* configuration of the tropinone aldol was also confirmed by single-crystal diffraction. Derivatization of isomeric tropinone and granatanone aldols as TBDMS ethers combined with their isomerization in the presence of silica can be used for determination of the relative configurations of these types of compounds.

## Experimental

Thin-layer chromatography (TLC) was performed on precoated plates (Merck, silica gel 60, F_254_). The spots were detected by using UV light (254 nm) and with phosphomolybdic acid followed by charring. Magnetic resonance spectra (^1^H NMR and ^13^C NMR) were recorded on a Bruker AVANCE II 400 spectrometer in CDCl_3_ at ambient temperature. Chemical shifts are reported in parts per million (ppm) downfield of tetramethylsilane. All reagents were purchased from Aldrich. Granatanone was sublimed; benzaldehyde was purified by standard techniques [29]. Dry triethylamine (Et_3_N) and dichloromethane (DCM) were distilled from calcium hydride. All air-sensitive reactions were carried out under argon.

Crystallographic data were collected with a Bruker-Nonius Apex-X8 CCD-diffractometer with Cu Kα radiation (λ = 1.54178 Å) at 296 K. The structure was solved by direct methods and refined by using the *SHELXS97* [30] and *SHELXL97* [30] programs. All non-H atoms were refined anisotropically; all H atoms bonded to carbon atoms were placed on geometrically calculated positions and refined by using a riding model. The hydroxy H atom in the structure was located from Δρ maps and refined isotropically. Crystallographic data (excluding structure factors) for the structure in this paper have been deposited with the Cambridge Crystallographic Data Centre as supplementary publication no. CCDC 887610, for compound *exo*,*syn*-**3**. Copies of the data can be obtained, free of charge, on application to CCDC, 12 Union Road, Cambridge CB2 1EZ, UK, (fax: +44-(0)1223-336033 or e-mail: deposit@ccdc.cam.ac.uk).

***exo*,*anti*-2-[Hydroxy(phenyl)methyl]-8-methyl-8-azabicyclo[3.2.1]octan-3-one (*exo*,*anti*-3)** [3–4,12] Colorless solid; mp 118–121 °C (decomp.); ^1^H NMR δ 7.32–7.25 (m, 5H), 5.23 (d, *J* = 3.1 Hz, 1H), 3.61–3.59 (m, 1H), 3.50–3.47 (m, 1H), 2.86 (ddd, *J* = 15.6 Hz, 4.6 Hz, 1.5 Hz, 1H), 2.47 (s, 3H), 2.45–2.42 (m, 1H, eq-H at C-2), 2.33 (dt, *J* = 15.6 Hz, 1.9 Hz, 1H), 2.21–2.12 (m, 1H), 1.69–1.53 (m, 1H); *R*_f_ 0.50 (10% MeOH/DCM).

***exo*,*syn*-2-[Hydroxy(phenyl)methyl]-8-methyl-8-azabicyclo[3.2.1]octan-3-one (*exo*,*syn*-3)** [20] Colorless solid; mp 81–83 °C (decomp.); ^1^H NMR δ 7.47–7.22 (m, 5H), 7.35 (br s, 1H), 5.01 (d, *J* = 2.6 Hz, 1H), 3.51–3.43 (m, 1H), 3.25–3.18 (m, 1H), 2.98 (ddd, *J* = 17.0 Hz, 5.2 Hz, 1.7 Hz, 1H), 2.43 (dt, *J* = 17.0 Hz, 1.7 Hz, 1H), 2.39–2.37 (m, 1H, eq-H at C-2), 2.37 (s, 3H), 2.20–2.03 (m, 2H), 1.70–1.61 (m, 1H), 1.42–1.35 (m, 1H); ^13^C NMR δ 210.7, 143.7, 128.3, 126.9, 125.5, 75.7, 63.1, 61.2, 50.3, 40.4, 26.8, 26.4; HRMS–ESI (*m*/*z*): [M + Na]^+^ calcd for C_15_H_19_NO_2_Na, 268.1313; found, 268.1325; *R*_f_ 0.40 (10% MeOH/DCM, decomp.). The crystal structure has been deposited at the Cambridge Crystallographic Data Centre and allocated the deposition number CCDC 887610; unit-cell parameters: *a* = 6.1202(1), *b* = 16.9100(3), *c* = 12.6953(2) Å, β = 91.1250(10) space group *P*2_1_/*n*.

***exo*,*anti*-2-[(*tert*-Butyldimethylsilyloxy)(phenyl)methyl]-8-methyl-8-azabicyclo[3.2.1]octan-3-one (*exo*,*anti*-5)** [3] The aldol *exo*,*anti-***3** (0.982 g, 3.97 mmol) was dissolved in dry DCM (11 mL). DMAP (0.057 g, 0.46 mmol) and dry Et_3_N (5.7 mL) were added, followed by the addition of TBDMSCl (1.193 g, 7.9 mmol). The resulting solution was allowed to stand at rt for 16 h. The reaction mixture was then diluted with DCM (5 mL), shaken with 20% aq K_2_CO_3_ (10 mL) and extracted with DCM (3 × 10 mL). The combined organic extracts were dried (Na_2_SO_4_), the solvent was removed under vacuum, and the residue was subjected to flash chromatography in hexanes/AcOEt (1:9), which gave the pure product as a colorless oil (1.283 g, 89%). ^1^H NMR δ 7.50–7.20 (m, 5H), 5.28 (d, *J* = 9.6 Hz, 1H), 3.39–3.37 (m, 1H), 2.87–2.82 (m, 1H, ax-H at C-4), 2.64–2.63 (m, 1H, *H*C-NMe), 2.36–2.33 (m, 1H, eq-H at C-2), 2.22 (s, 3H), 2.05–1.88 (m, 3H), 1.62–1.53 (m, 1H), 1.40–1.32 (m, 1H), 0.78 (s, 3H), −0.06 (s, 3H), −0.31(s, 3H); ^13^C NMR δ 209.4, 143.3, 128.1, 127.6, 127.1, 75.3, 68.6, 63.7, 63.0, 49.0, 41.1, 25.71, 25.65, 25.5, 17.9, −4.7, −5.2.

***exo*,*syn*-2-[(*tert*-Butyldimethylsilyloxy)(phenyl)methyl]-8-methyl-8-azabicyclo[3.2.1]octan-3-one (*exo*,*syn*-5)** Product *exo*,*syn*-**5** was obtained as a yellow oil (0.390 g, 94%) in the same way as *exo*,*anti*-**5** by using *exo*,*syn*-**3** (0.283 g, 1.15 mmol), dry DCM (3.3 mL), DMAP (0.016 g, 0.13 mmol), dry Et_3_N (1.6 mL), and TBDMSCl (0.344 g, 2.28 mmol). Attempted flash chromatographic purification on silica led to significant isomerization. ^1^H NMR δ 7.38–7.16 (m, 5H), 5.24 (d, *J* = 9.3 Hz, 1H), 3.68–3.62 (m, 1H), 3.42–3.35 (m, 1H), 2.57 (ddd, *J* = 14.7 Hz, 4.4 Hz, 1.9 Hz, 1H), 2.45 (dt, *J* = 9.3 Hz, 1.9 Hz, 1H, eq-H at C-2), 2.41 (s, 3H), 2.25–2.05 (m, 3H), 1.60–1.48 (m, 2H), 0.87 (s, 9H), 0.10 (s, 3H), −0.26 (s, 3H); ^13^C NMR δ 209.2, 142.5, 128.1, 127.7, 126.5, 74.1, 69.7, 63.3, 62.9, 50.7, 41.3, 18.1, 26.0, 25.8, 25.8, −4.51, −4.96; HRMS–ESI (*m*/*z*): [M + Na]^+^ calcd for C_21_H_33_NO_2_SiNa, 382.2178; found, 382.2161; *R*_f_ 0.75 (40% AcOEt/hexanes).

***endo*,*syn*-2-[(*tert*-Butyldimethylsilyloxy)(phenyl)methyl]-8-methyl-8-azabicyclo[3.2.1]octan-3-one (*endo*,*syn*-5)** [3] The TBDMS-ether *exo*,*anti*-**5** (0.179 g, 0.50 mmol) was applied on a flash silica column in hexanes/AcOEt (9:1) and left for 18 h. The column was washed with hexanes/AcOEt (1:1) to remove the starting material (0.030 g, 16%) and with MeOH/DCM (1:9) to remove the product of isomerization as an oil (0.141 g, 79%). ^1^H NMR δ 7.39–7.21 (m, 5H), 5.10 (d, *J* = 7.3 Hz), 3.74–3.71 (m, 1H), 3.48–3.38 (m, 1H, *H*C-NMe), 3.04–2.96 (m, 1H, ax-H at C-2), 2.68–2.58 (m, 1H, ax-H at C-4), 2.48 (s, 3H), 2.12–1.93 (m, 4H), 1.63–1.54 (m, 1H), 0.85 (s, 9H), 0.04 (s, 3H), −0.31 (s, 3H); ^13^C NMR δ 208.1, 144.4, 127.8, 127.3, 127.0, 71.3, 63.0, 62.0, 61.4, 47.7, 38.0, 27.7, 25.8, 25.6, 24.4, 18.0, −4.6, −5.3.

***endo*,*anti*-2-[(*tert*-Butyldimethylsilyloxy)(phenyl)methyl]-8-methyl-8-azabicyclo[3.2.1]octan-3-one (*endo*,*anti*-5)** The TBDMS-ether *exo*,*syn*-**5** (0.140 g, 0.39 mmol) was applied on a flash silica column in hexanes/AcOEt (9:1). The column was washed with hexanes/AcOEt (1:1) to remove the starting material (0.028 g, 20%) and with MeOH/DCM (1:9) to remove the product of isomerization as a colorless oil (0.082 g, 59%). ^1^H NMR δ 7.26–7.19 (m, 5H), 5.01 (d, *J* = 7.6 Hz, 1H), 3.43–3.37 (m, 1H), 3.06 (ddd, *J* = 7.6 Hz, 1.5 Hz, 1.5 Hz, 1H ax-H at C-2), 2.80–2.74 (m, 2H), 2.40 (s, 3H), 2.14 (dd, *J* = 13.9 Hz, 2.4 Hz, 1H), 1.95–1.85 (m, 1H), 1.65–1.46 (m, 2H) 1.38–1.28 (m, 1H), 0.78 (s, 9H), 0.09 (s, 3H), −0.16 (s, 3H); ^13^C NMR δ 207.8, 142.5, 128.2, 127.7, 127.0, 71.6, 63.6, 62.4, 61.5, 47.2, 37.0, 27.7, 25.7, 24.7, 18.1, −4.83, −4.90; HRMS–ESI (*m*/*z*): [M + Na]^+^ calcd for C_21_H_33_NO_2_SiNa, 382.2178; found, 382.2191; *R*_f_ 0.25 (40% AcOEt/hexanes).

## Supporting Information

File 1 Experimental procedures for the preparation and characterization of the remaining compounds.
