# Supplementary material for: Determination of the relative configuration of tropinone and granatanone aldols by using TBDMS ethers
Source: Beilstein J Org Chem. 2012 Nov 2;8:1877–83. doi: 10.3762/bjoc.8.216 (PMC3511025; doi:10.3762/bjoc.8.216)
Supplement: File 1 — Experimental procedures for the preparation and characterization of the remaining compounds. [file Beilstein_J_Org_Chem-08-1877-s001.pdf]

Supporting Information

for

Determination of the relative configuration of tropinone and granatanone aldols by using TBDMS ethers

Ryszard Lazny*[1], Aneta Nodzewska[1], Katarzyna Sidorowicz[1], Przemyslaw Kalicki[2]

Address: [1]Institute of Chemistry, University of Bialystok, ul. Hurtowa 1, 15-339 Bialystok, Poland and [2]Institute of Organic Chemistry, Polish Academy of Sciences, ul. Kasprzaka 44/52, 01-224 Warsaw, Poland

Email: Ryszard Lazny* - lazny@uwb.edu.pl

* Corresponding author

Experimental procedures for the preparation and characterization of the remaining compounds

***exo*,*anti*-2-[Hydroxy(phenyl)methyl]-9-methyl-9-azabicyclo[3.3.1]nonan-3-one (*exo*,*anti*-4)** [1] Mp 103–106 °C; R_f 0.38 (10% MeOH/DCM); ^{1}H NMR δ 7.72 (br s, 1H), 7.36-7.30 (m, 5H), 5.27 (d, *J* = 3.6 Hz, 1H), 3.38 (d, *J* = 4.1 Hz, 1H), 3.35-3.28 (m, 1H), 2.98 (dd, *J* = 16.2 Hz, 7.0 Hz, 1H), 2.74 (s, 3H), 2.57 (d, *J* = 3.8 Hz, 1H), 2.45 (d, *J* = 16.2 Hz, 1H), 2.20-2.05 (m, 2H), 1.61-1.52 (m, 2H), 1.38-1.29 (m, 2H).

Typical procedure for preparation *exo*,*syn* aldols:

***exo*,*syn*-2-[Hydroxy(phenyl)methyl]-9-methyl-9-azabicyclo[3.3.1]nonan-3-one (*exo*,*syn*-4)** [1] Benzaldehyde (0.102 mL, 1 mmol) was added to a mixture of granatanone (0.306 g, 2 mmol) and water (0.011 mL, 0.61 mmol). The reaction mixture was then stirred at room temperature until NMR monitoring showed satisfactory conversion (33 d – conversion 30%). The mixture was taken up in toluene and evaporated under vacuum (repeated 3 times). The residue was crystallized from cyclohexane or diethyl ether to give *exo*,*syn*-**4** (0.047 g, 18%). Mp 117–118 °C (decomp.); R_f 0.46 (7% MeOH/DCM); ^{1}H NMR δ 7.62 (br s, 1H), 7.44-7.22 (m, 5H), 5.08 (d, J = 2.0 Hz, 1H), 3.34-3.28 (m, 1H), 3.17 (dd, J = 17.1 Hz, 7.2 Hz, 1H), 3.05 (d, J = 4.2 Hz, 1H), 2.64 (s, 3H), 2.54 (d, J = 17.1 Hz, 1H), 2.48 (s, 1H), 2.12-2.01 (m, 1H), 1.97-1.88 (m, 1H), 1.55-1.42 (m, 2H), 1.36-1.29 (m, 1H), 1.01-0.91 (m, 1H); ^{13}C NMR δ 211.6, 143.7, 128.2, 126.8, 125.4, 76.1, 60.0, 53.6, 53.5, 46.9, 39.6, 22.6, 22.3, 16.4; HRMS (ESI): 282.1465 [M^+ + H] calcd for $C_{16}H_{21}NO_2Na$, 282.1470.

***exo*,*anti*-2-[(*tert*-Butyldimethylsilyloxy)(phenyl)methyl]-9-methyl-9-azabicyclo[3.3.1]nonan-3-one (*exo*,*anti*-6)** The aldol *exo*,*anti*-**4** (0.259 g, 1 mmol) was dissolved in dry DCM (3 mL). DMAP (0.013 g, 0.11 mmol) and dry Et_3N (1.4 mL) were added, followed by the addition of TBDMSCl (0.300 g, 2 mmol). The resulting solution was allowed to stand at rt for 16 h. The reaction mixture was then diluted with DCM, shaken with 20% aq. K_2CO_3 and extracted with DCM (3 × 10 mL). The combined organic extracts were dried (Na_2SO_4), the solvent was removed under vacuum, and the residue was subjected to flash chromatography in hexanes/AcOEt (1:9), which gave the pure product as a white solid (0.306 g, 82%). Mp 59–60°°C (decomp.); R_f 0.57

(MeOH/DCM); ^{1}H NMR δ 7.40-7.25 (m, 5H), 5.21 (d, J = 9.3 Hz, 1H), 3.27-3.20 (m, 1H), 3.01-2.89 (m, 1H), 2.56 (dd, J = 16.2 Hz, 7.0 Hz, 1H), 2.52 (s, 3H), 2.49-2.40 (m, 1H, eq-H at C-2), 2.33 (d, J = 15.2 Hz, 1H), 2.08-1.96 (m, 1H), 1.94-1.83 (m, 1H), 1.74-1.62 (m, 1H), 1.49-1.41 (m,1H), 1.31-1.21 (m, 1H), 1.01-0.91 (m,1H) 0.81 (s, 9H), -0.03 (s, 3H), -0.33 (s, 3H); ^{13}C NMR δ 211.2, 143.6, 128.1, 128.0, 127.7, 76.1, 65.4, 55.6, 55.4, 46.1, 40.4, 25.6, 22.4, 22.2, 18.0, 17.0, −4.6, −5.2; HRMS (ESI): 396.2350 [M^{+} + Na] calcd for $C_{22}H_{35}NO_2SiNa$, 396.2335.

***exo*,*syn*-2-[(*tert*-Butyldimethylsilyloxy)(phenyl)methyl]-9-methyl-9-azabicyclo[3.3.1]nonan-3-one (*exo*,*syn*-6)** Product *exo*,*syn*-**6** was obtained as a white solid (0.243 g, 65%) in the same way as *exo*,*anti*-**6** by using *exo*,*syn*-**4** (0.259 g, 1 mmol), dry DCM (3 mL), DMAP (0.013 g, 0.11 mmol), Et_3N (1.4 mL) and TBDMSCl (0.300 g, 2 mmol). Mp 77–78 °C (decomp.); R_f 0.57 (7% MeOH/DCM); ^{1}H NMR δ 7.45-7.18 (m, 5H), 5.16 (d, J = 8.6 Hz, 1H), 3.44 (d, J = 4.0 Hz 1H), 3.27-2.20 (m, 1H), 2.68 (s, 3H), 2.64 (d, J = 6.9 Hz,1H), 2.54 (s, 1H, eq-H at C-2), 2.20 (dt, J = 16.9 Hz,1.3 Hz, 1H), 2.14-1.95 (m, 2H), 1.60-1.42 (m, 2H), 1.30-1.12 (m, 2H), 0.85 (s, 9H), 0.05 (s, 3H), −0.28 (s, 3H); ^{13}C NMR δ 211.0, 142.5, 128.0, 127.6, 126.6, 106.6, 74.8, 66.2, 55.2, 47.3, 40.8, 25.8, 23.1, 22.9, 18.1, 17.0, −4.6, −5.0; HRMS (ESI): 396.2354 [M^{+} + Na] calcd for $C_{22}H_{35}NO_2SiNa$, 396.2335.

***endo*,*syn*-2-[(*tert*-Butyldimethylsilyloxy)(phenyl)methyl]-9-methyl-9-azabicyclo[3.3.1]nonan-3-one (*endo*,*syn*-6)** The TBDMS-ether *exo*,*anti*-**6** (0.030 g, 0.08 mmol) was applied on a flash silica column in DCM. The column was washed with MeOH/DCM (4:96) to remove the product of isomerization as a colorless oil (0.023 g, 77%). R_f 0.54 (7% MeOH/DCM); ^{1}H NMR δ 7.40-7.20 (m, 5H), 5.26 (d, J = 6.8 Hz, 1H),

3.43-3.38 (m, 1H), 3.29-3.23 (m, 1H), 3.03-2.91 (m, 1H, ax-H at C-2), 2.70 (dd, J = 15.4 Hz, 6.7 Hz, 1H), 2.58 (s, 3H), 2.18 (d, J = 15.5 Hz, 1H), 2.07-1.89 (m, 2H), 1.87-1.75 (m, 1H), 1.59-1.46 (m, 3H), 0.84 (s, 9H), 0.09 (s, 3H), -0.36 (s, 3H); ^{13}C NMR δ 209.4, 144.2, 127.9, 127.2, 127.1, 71.4, 58.0, 56.7, 56.2, 43.0, 41.0, 29.4, 25.5, 22.4, 18.1, 16.5, −4.6, −5.3; HRMS (ESI): 396.2344 [M^+ + Na] calcd for $C_{22}H_{35}NO_2SiNa$, 396.2335.

***endo*,*anti*-2-[(*tert*-Butyldimethylsilyloxy)(phenyl)methyl]-9-methyl-9-azabicyclo[3.3.1]nonan-3-one (*endo*,*anti*-6)** The TBDMS-ether *exo*,*syn*-**6** (0.030 g, 0.08 mmol) was applied on a flash silica column in DCM. The column was washed with MeOH/DCM (2:98) to remove the product of isomerization as an oil (0.024 g, 80%). R_f 0.45 (7% MeOH/DCM); ^{1}H NMR δ 7.45-7.20 (m, 5H), 5.01 (d, J = 8.7 Hz, 1H), 3.29-3.24 (m, 1H), 3.14 (dd, J = 8.5 Hz, 5.2 Hz, 1H, ax-H at C-2), 2.85 (dd, J = 14.6 Hz, 6.7 Hz, 1H), 2.55 (s, 3H), 2.52 (s, 1H), 2.26 (dd, J = 14.5 Hz, 0.9 Hz, 1H), 2.11 (m, 1H), 1.89-1.78 (m, 1H), 1.65-1.56 (m, 2H), 1.54-1.47 (m, 2H), 0.72 (s, 9H), 0.08 (s, 3H), −0.15 (s, 3H); ^{13}C NMR δ 210.0, 142.0, 128.2, 127.5, 125.5, 72.1, 59.5, 57.6, 56.0, 47.3, 43.1, 40.8, 30.2, 25.7, 18.1, 16.0, −4.9, −5.1; HRMS (ESI): 396.2315 [M^+ + Na] calcd for $C_{22}H_{35}NO_2SiNa$, 396.2335.

Reference

1. Lazny, R.; Wolosewicz, K.; Zielinska, P.; Urbanczyk-Lipkowska, Z.; Kalicki, P. *Tetrahedron* **2011**, *67*, 9433-9439.
